# Gender difference of emotional bias in sharing love

**DOI:** 10.1186/1471-2202-12-S1-P328

**Published:** 2011-07-18

**Authors:** David Nicoladie Tam

**Affiliations:** 1 Department of Biological Sciences, University of North Texas, Denton, TX 76203, USA

## 

Having developed a computational model for emotional response (Emotional-Gain Model) [1-4] and a model for fairness (Fairness-Equity Model) [5] that quantified emotional bias and fairness bias, we will address the gender difference between the perception of love. We employed the experimental paradigm called “ultimatum game” to elicit emotional responses to the sharing of love and money. The experimental paradigm essentially asks human subjects to split a sum of money or love such that their emotional response to fairness can be assessed. The results showed that although male and female respond very similarly in their emotional perception and fairness perception, except for the subtle difference as revealed by our Emotional-Gain and Fairness-Equity Models. The results revealed that female tends to recognize the full spectrum of emotions (both positive and negative) while male tends to recognize the positive emotions more often than the negative emotions. Furthermore, female tends to value love much more important than money, whereas male tends to value money more than love at a subconscious level. Both genders are consciously aware of the objectivity but responded subjectively to what they considered as important, with the value system emphasizing more on money for male whereas the value system emphasizes more on love for female. This confirms the emotional bias and fairness bias quantitative by the emotional and fairness models experimentally.
